# Angiopoietin-Like Proteins: A Comprehensive Look

**DOI:** 10.3389/fendo.2014.00004

**Published:** 2014-01-23

**Authors:** Gaetano Santulli

**Affiliations:** ^1^Department of Advanced Biomedical Sciences, University of Naples “Federico II”, Naples, Italy; ^2^College of Physicians and Surgeons, Columbia University Medical Center, New York, NY, USA

**Keywords:** angiopoietin-like proteins, ANGPTL, metabolism, hematopoietic stem cells, cancer, glucose homeostasis, insulin, inflammation

## Abstract

Angiopoietin-like proteins (ANGPTLs) are a family of proteins structurally similar to the angiopoietins. To date, eight ANGPTLs have been discovered, namely ANGPTL1 to ANGPTL8. Emerging evidence implies a key role for ANGPTLs in the regulation of a plethora of physiological and pathophysiological processes. Most of the ANGPTLs exhibit multibiological properties, including established functional roles in lipid and glucose metabolism, inflammation, hematopoiesis, and cancer. This report represents a systematic and updated appraisal of this class of proteins, focusing on the main features of each ANGPTL.

## Angiopoietin-Like Proteins

A family of proteins that is structurally similar to the angiopoietins has been identified as angiopoietin-like proteins (ANGPTLs). To date, eight ANGPTLs have been discovered, namely ANGPTL1 to ANGPTL8 (Table 1). All ANGPTLs contain an amino-terminal coiled-coil domain, a linker region, and a carboxy-terminal fibrinogen-like domain. ANGPTL8 is the exception of the family, since there is no fibrinogen-like domain.

**Table 1:** **Angiopoietin-like proteins**.

ANGPTL	Other common names	Main tissue expression	Chromosome (human)	Chromosome (mouse)	Reference
1	ARP1, angioarrestin, ANG3, AngY, ANGPT3, UNQ162, dJ595C2.2, 2810039D03Rik	Liver, muscle	1	1	Dhanabal et al. (1)
2	ARP2, AI593246, AW260363, HARP	Heart, vessels, adipose tissue	9	2	Doi et al. (2)
3	ANL3, ANG-5, FHBL2, ANGPT5	Liver	1	4	Glazer (3)
4	ARP4, PGAR, HFARP, FIAF	Liver, Adipose tissue, brain, intestine, thyroid, kidney, heart	19	7	Ortega-Senovilla et al. (4)
5	ANGL5_HUMAN, A_14_P125422, NP835228.1	Heart	11	–	Zeng et al. (5)
6	AGF, ARP5, ARP3	Liver	19	9	Oike et al. (6)
7	AngX, dJ647M16.1, CDT6, RP4-647M16.2	Eye (trabecular meshwork)	1	4	Peek et al. (7)
8	Lipasin, betatrophin, TD26, c19orf80, RIFL PRO1185, PVPA599, Gm6484	Liver, adipose tissue	19	9	Quagliarini et al. (8), Fu et al. (9)

*ARP, angiopoietin-related protein, PGAR, PPARγ-angiopoietin-related, HFARP, hepatic fibrinogen angiopoietin-related protein, FIAF, fasting-induced adipose factor; RIFL, re-feeding induced in fat and liver*.

Angiopoietin-like proteins have been generally considered orphan ligands as they do not bind to the receptors classically targeted by angiopoietins (10, 11), namely the tyrosine kinase with immunoglobulin-like and EGF-like domain 1 (Tie1) and the endothelial-specific receptor tyrosine kinase (TEK or Tie2). This aspect, which constitutes the main difference between angiopoietins and ANGPTLs, indicates that the functional mechanism of ANGPTL proteins may be different from that of angiopoietins (12). However, numerous studies show that ANGPTL proteins, similar to angiopoietins, are able to potently regulate angiogenesis (12–14).

Interestingly, Zheng and colleagues have recently shown that the immune-inhibitory receptor human leukocyte immunoglobulin-like receptor B2 (LILRB2) and its mouse ortholog paired immunoglobulin-like receptor (PIRB) are receptors for angptl1, 2, 5, and 7 (15). Such a study indicates an unexpected functional significance of classical immune-inhibitory receptors in the maintenance of stemness of normal adult stem cells and in support of cancer development (15).

Various functions of ANGPTL proteins have also been described in developmental, physiological, and pathophysiological processes. Crucially, some ANGPTL proteins exhibit multibiological properties, including functional roles in lipid metabolism (16), inflammation (17), hematopoietic stem cell activity (18), and cancer cell invasion (19, 20).

## ANGPTL1

ANGPTL1 is the first member of the ANGPTL family discovered (10) and is considered to be a potent regulator of angiogenesis. In particular, it has been reported as a key anti-angiogenic protein (it is also known as angioarrestin) by inhibiting the proliferation, migration, tube formation, and adhesion of endothelial cells (1). As well as being anti-angiogenic, ANGPTL1 has also been shown to exhibit antiapoptotic activity in human endothelial cells by stimulating phosphorylation of ERK 1/2 and Akt-1 (21).

Growing evidence suggests that ANGPTL proteins not only target endothelial cells but also affect tumor cell behavior. Indeed, ANGPTL1 transcript has been found to be down-regulated in several tumor specimens, including lung, prostate, kidney, thyroid, and urinary bladder cancers (1). Consequently, inhibition of tumor growth and metastasis has been proposed by independent investigators as one of the major effects of ANGPTL1 (22, 23). Of note, mouse *Angptl1* shares 75% nucleotide identity and 92% amino acid identity (456/491 residues in product) with human *Angptl1*, suggesting evolutionary conservation and functional homology (24). In a screen of 102 patients with lung cancer, ANGPTL1 expression was found to be inversely correlated with invasion, lymph-node metastasis, and poor clinical outcomes (20). ANGPTL1 suppressed the migratory, invasive, and metastatic capabilities of lung and breast cancer cell lines *in vitro* and reduced metastasis *in vivo* (mice injected with cancer cell lines overexpressing ANGPTL1). Ectopic expression of ANGPTL1 inhibited the epithelial-to-mesenchymal transition by inducing expression of microRNA-630 and subsequently reducing the expression of the zinc-finger protein SLUG (20). Ergo, there is clinical evidence that ANGPTL1 expression inversely correlates with advanced-stage lymph-node metastasis and positively correlates with survival of patients with cancer.

## ANGPTL2

ANGPTL2 is a circulating glycoprotein with abundant expression in the heart, adipose tissue, lung, kidney, and skeletal muscle. Its expression is stimulated by hypoxia and induces angiogenesis and endothelial cell migration. It may exert a function on endothelial cells through autocrine or paracrine action (25–27). Circulating levels of ANGPTL2 correlate with inflammation, adiposity, and insulin resistance in both mice and humans (17). An increase in mRNA levels of ANGPTL2 has been reported in endothelial cells isolated from arteries of active smokers with severe coronary artery disease compared with non-smokers (28). Moreover, ANGPTL2 has recently been associated with chronic inflammation in dermatomyositis (29), synovial inflammation in rheumatoid arthritis (30), abdominal aortic aneurysms (31), and cancer (32–34). In addition, ANGPTL2 has recently been shown to causally contribute to the development of chronic endothelial/vascular inflammation leading to atherosclerosis (35). Muramoto and coworkers evaluated serum ANGPTL2 levels in overweight men after lifestyle intervention, providing evidence that ANGPTL2 is a highly sensitive indicator of reduced visceral fat and metabolic improvement (36). In a recent study conducted in a general community-dwelling Japanese population, elevated serum ANGPTL2 levels were positively associated with the development of type 2 diabetes mellitus independent of other risk factors including C-reactive protein (CRP) levels (2). Constitutive ANGPTL2 activation *in vivo* induces inflammation of the vasculature characterized by adhesion of leukocytes to the vessel walls and increased permeability. ANGPTL2 deletion has also been shown to ameliorate adipose tissue inflammation and systemic insulin resistance in diet-induced obese mice (17). On the other hand, Hashimoto and colleagues demonstrated *in vitro* that replenishment of Angptl2 is able to stimulate insulin sensitivity and improves the type 2 diabetic state in murine adipocytes (37). Given the significant correlation between ANGPTL2 levels and insulin resistance (17, 36) and the recent evidence of its pro-oxidant effect (34), ANGPTL2 could also be useful as a biomarker of endothelial dysfunction. In summary, ANGPTL2 can be considered a key mediator that links obesity to systemic insulin resistance and plays a pivotal role in the atherosclerotic process and in the development of diabetes.

## ANGPTL3

Angiopoietin-like 3 (ANGPTL3) is a main regulator of lipoprotein metabolism. Its function is at least in part linked to the inhibition of lipoprotein lipase (LPL) activity (38, 39). ANGPTL3 is activated by cleavage at a proprotein convertase consensus site to release the N-terminal domain and its activity is regulated by ANGPTL8 (8, 38). Of note, ANGPTL3-deficient subjects have reduced cholesterol levels in all major plasma lipoprotein fractions and a marked reduction of triglycerides, especially in very low-density (VLDL) and high-density (HDL) lipoproteins (40, 41). Loss-of-function mutations in ANGPTL3 have been shown to cause familial combined hypolipidemia (3, 42–45).

ANGPTL3-deficient mice exhibit low plasma HDL cholesterol and HDL phospholipid (PL), which were increased by ANGPTL3 supplementation via adenovirus (46). *In vitro*, ANGPTL3 inhibited the phospholipase activity of endothelial lipase (EL), which hydrolyzes HDL-PL and consequently decreases plasma HDL levels, through a putative heparin-binding site in the N-terminal domain of ANGPTL3 (46).

In humans, the complete absence of ANGPTL3 results in an increased LPL activity and mass and low circulating free fatty acid levels (41). This latter effect is probably due to a decreased mobilization of free fatty acid from fat stored in human adipose tissue and may result in reduced hepatic VLDL synthesis and secretion via attenuated hepatic free fatty acid supply. ANGPTL3 may also affect insulin sensitivity, playing a major role in modulating both lipid and glucose metabolism (41). In addition to that, ANGPTL3 has been shown *in vivo* to be negatively regulated by thyroid hormone (47) and serum ANGPTL3 levels have been reported to be significantly higher in patients with rheumatic disorders, including dermatomyositis and systemic sclerosis (48). A potential role for ANGPTL3 has also been proposed in the pathogenesis of atherosclerosis, since its levels have been shown to be closely associated with arterial wall thickness (49).

## ANGPTL4

ANGPTL4 is involved in a variety of functions, including lipoprotein metabolism and angiogenesis (50, 51). Growing evidence indicates that ANGPTL4 serves as a potent inhibitor of the LPL enzyme, which hydrolyzes triglycerides from the apolipoprotein B – containing lipoproteins chylomicrons and VLDL (52). Through means of such a mechanism, ANGPTL4 suppresses the release of non-esterified fatty acids (NEFAs) and their subsequent uptake by underlying tissues, including adipose tissue, skeletal and cardiac muscle. Moreover, ANGPTL4 increases the intracellular lipolysis of triglycerides within adipocytes, thereby raising plasma NEFA concentrations (53, 54). The expression of ANGPTL4 has been shown to be governed through a synergistic induction by the lipid-sensing peroxisome proliferator-activated receptors (PPARs) α, β, and γ (53). ANGPTL4 overexpression causes a 50% reduction in adipose tissue weight by stimulating lipolysis, fatty acids oxidation, and uncoupling in fat (53–55).

Of interest, insulin has been shown to induce a decrease in ANGPTL4 plasmatic levels (56). During a hyperinsulinemic euglycemic clamp in healthy subjects, insulin induced a reduction in plasma ANGPTL4 and NEFA concentrations and decreased ANGPTL4 mRNA in adipose tissue (56). In a recent report, the change in ANGPTL4 was shown to positively correlate with the change in NEFA concentrations and negatively correlate with the change in plasma triglycerides (52). The effect of insulin on plasma ANGPTL4 could be thereby mediated by the observed decrease in NEFA. However, other reports indicate that insulin can suppress ANGPTL4 production independent of NEFA (57, 58). Recent studies by Clement and colleagues elegantly demonstrated, both in humans and in animal models, that ANGPTL4 is a direct molecular link between proteinuria and hypertriglyceridemia in nephrotic syndrome (59–61).

ANGPTL4 also seems to play a relevant role in type 2 diabetes mellitus and in the metabolic syndrome, both associated with dyslipidemia (52). In mice, ANGPTL4 decreases blood glucose and improves glucose tolerance but induces hyperlipidemia and hepatic steatosis (62). Central administration of Angptl4 suppresses food intake and body weight gain via suppression of hypothalamic AMPK activities (63). A recent report suggested a role for ANGPTL4 also in the pathophysiology of atherosclerosis. Indeed, a microarray analysis demonstrated that in high-calcified carotid plaques ANGPTL4 expression was significantly elevated, whereas FGFR2 expression was significantly suppressed (64).

ANGPTL4 and ANGPTL3 share many common features. However, while ANGPTL4 exhibits a widespread distribution of tissue expression, ANGPTL3 is almost exclusively expressed in the liver (8, 65, 66). Besides, whereas ANGPTL3 inhibits LPL activity primarily in the fed state, ANGPTL4 plays important roles in both fed and fasted states. ANGPTL4 regulates the tissue-specific delivery of lipoprotein-derived fatty acids. Differently from ANGPTL3, ANGPTL4 is thus considered an endocrine or autocrine/paracrine inhibitor of LPL depending on its sites of expression (67). Treatments with various ligands of nuclear receptors revealed that ANGPTL3 is a target gene of liver X receptor, while ANGPTL4 expression is activated by ligands of all PPARs. Thus, the differential regulation of ANGPTL3 and ANGPTL4 by sites of expression, nutritional status, and ligands of nuclear receptors may confer unique roles of each in lipoprotein metabolism (68).

## ANGPTL5

During large-scale DNA sequencing of the human fetal brain cDNA library, a novel human angiopoietin-like cDNA was cloned (5) and termed human angiopoietin-like 5 (ANGPTL5). No mouse ortholog has been described hitherto. Like other members of the angiopoietin family, ANGPTL5 protein also has an N-terminal cleavable signal peptide, a predicted coiled-coil domain, and a fibrinogen-like domain. The search against the human genome database indicates that ANGPTL5 maps to 11q22. Expression analysis of ANGPTL5 shows that it is mainly expressed in adult human heart (5). Most recently, it has been proved to play a functional role in the expansion of human cord blood hematopoietic stem cells (69, 70). Of interest, other ANGPTLs have been shown to participate in signaling pathways for the survival as well as expansion of human hematopoietic stem cells in the bone-marrow niche (71).

## ANGPTL6

Serum ANGPTL6 levels have been shown to be significantly higher in patients with metabolic syndrome compared with healthy subjects (72). Moreover, among the components of metabolic syndrome, subjects with high waist circumference or decreased HDL cholesterol had significantly increased serum ANGPTL6 levels (72). These findings seem somehow in contrast with the previous demonstration of a functional role of ANGPTL6 in counteracting obesity and related insulin resistance (73). Further investigations in this field are therefore warranted. In another study, four single nucleotide polymorphisms (SNPs: rs6511435, rs8112063, rs11671983, and rs15723) were found to cover more than 95% of the known ANGPTL6 genetic variability. Subjects from the entire “MONICA-Study” were genotyped for these SNPs (74). The G allele of rs8112063 was associated with lower plasma glucose levels. Furthermore, the G allele of rs6511435 tended to be associated with a 20% higher risk of metabolic syndrome and obese patients carrying such allele had significantly higher plasma insulin levels than AA subjects (*P* = 0.0055). Instead, no significant association was detected for rs11671983 and rs15723 (74).

A potential role of ANGPTL6 in endothelial dysfunction is suggested by preliminary results from a recent Finnish study demonstrating that Angptl6 serum levels are higher in women with subsequent pregnancy-induced hypertension (75). Supporting these findings, higher values of resting metabolic rates have been found in subjects with higher circulating ANGPTL6 concentration (76). Interestingly, there was a significant difference in weight, body mass index, fat mass, visceral fat, fasting serum glucose and insulin, and CRP among those with different levels of the serum ANGPTL6 concentration (76).

## ANGPTL7

ANGPTL7 gene is located within intron 28 of *FRAP1* gene encoding mTOR protein (77) at human chromosome 1p36.22 (78). *In silico* expression analyses have been performed to compare the expression profiles of human and mouse ANGPTL7 mRNAs. Human ANGPTL7 mRNA was expressed in neural tissues, keratoconus cornea, trabecular meshwork, melanotic melanoma, and uterus endometrial cancer, while mouse mRNA was essentially expressed in four-cell embryo, synovial fibroblasts, thymus, uterus, and testis (78).

Albeit structurally related to the angiopoietins, ANGPTL7’s biological function is poorly understood. As a potent target gene of WNT/β-catenin signaling pathway, it is generally considered a potential target in the fields of regenerative medicine and oncology. Recently, a functional role for ANGPTL7 has been proposed in the pathophysiology of glaucoma (79, 80). Indeed, the concentration of ANGPTL7 protein was found to be elevated in aqueous humor from patients with glaucoma (79, 81). Furthermore, overexpression of ANGPTL7 in primary human trabecular meshwork cells altered the expression of relevant trabecular meshwork proteins of the extracellular matrix (ECM), including fibronectin, collagens type I, IV, and V, myocilin, versican, and MMP1. A dysfunctional trabecular meshwork leads to an altered fluid resistance, which results in increased intraocular pressure, the major risk factor of glaucoma, a leading cause of blindness in the developed world (81, 82). Interestingly, silencing ANGPTL7 during the glucocorticoid insult significantly affected the expression of other steroid-responsive proteins (79, 80).

## ANGPTL8

Also known as lipasin because of its capacity in LPL inhibition (9), this protein, ANGPTL8, is considered a novel but atypical ANGPTL family member, since it lacks the fibrinogen-like domain, the glycosylation sites, and the aminoacids for forming disulfide bonds. ANGPTL8 is a hepatocyte-derived circulating factor that regulates plasma triglycerides levels (8) and is thereby considered a key mediator of the post-prandial trafficking of fatty acids to adipose tissue. Mice deficient for ANGPTL8 have low triglyceride levels whereas these levels are increased after its adenovirus-mediated overexpression. Murine ANGPTL8 transcript is highly enriched in white and brown adipose tissue and liver (another way to refer to ANGPTL8 is actually RIFL, re-feeding induced in fat and liver). In adipocytes, ANGPTL8 is up-regulated by insulin and down-regulated by agents that stimulate lipolysis, including forskolin. Moreover, there is evidence of a roughly eightfold increase in ANGPTL8 transcript level in the adipose tissue of *ob/ob* mice compared with wild-type animals (83).

Lately, hepatic overexpression of ANGPTL8 has been shown to promote proliferation of pancreatic beta cells and increase insulin release in an insulin-deficient mouse model of insulin resistance (84). Hence, ANGPTL8 might certainly contribute to glucose homeostasis (85) opening a new door to possible future targeted therapies for diabetes and metabolic syndrome (86, 87).

## Conclusion

Numerous biological functions of ANGPTL proteins have been so far described both in physiological and pathophysiological processes. Some ANGPTL proteins have been shown to exhibit pleiotropic effects, including functional roles in metabolism, angiogenesis, inflammation, and cancer. In particular, the recent identification of mutations in distinct ANGPTLs is shedding light on the potential role of these proteins at the crossroads of lipoproteins, fatty acids, and glucose metabolism, thus making them attractive molecules to target the cardio-metabolic risk.

## Conflict of Interest Statement

The author declares that the research was conducted in the absence of any commercial or financial relationships that could be construed as a potential conflict of interest.
